# Advocacy of diagnostic criteria for maxillary incisive canal cysts based on alteration of normal maxillary incisive canals according to aging in Japanese populations

**DOI:** 10.1186/s13005-019-0209-5

**Published:** 2019-10-22

**Authors:** Naruhiko Ueda, Tatsurou Tanaka, Masafumi Oda, Nao Wakasugi-Sato, Shinobu Matsumoto-Takeda, Yuichi Miyamura, Takaaki Jyoujima, Kouichi Kiyota, Kensuke Tsutsumi, Yasuhiro Morimoto

**Affiliations:** Division of Oral and Maxillofacial Radiology, Kyushu Dental University, 2-6-1 Manazuru, Kokurakita-ku, Kitakyushu, 803-8580 Japan

**Keywords:** Computed tomography, Maxillary incisive canal, Aging, Incisive canal cysts

## Abstract

**Background:**

The purpose of the present study was to describe the CT imaging findings of normal incisive canals and incisive canal cysts and propose cut-off values to differentiate between them.

**Methods:**

A total of 220 normal subjects and 40 patients with incisive canal cysts on multi-detector row computed tomography (MDCT) were retrospectively analyzed. The shapes, sizes, anatomic variations, Hounsfield scale values, and so on of maxillary incisive canals and the sizes and Hounsfield scale values of maxillary incisive canal cysts were analyzed.

**Results:**

A significant difference in sizes of maxillary incisive canals in normal subjects was found between males and females. The sizes of maxillary incisive canals were significantly wider during aging, but shapes, anatomic variations, and Hounsfield scale values in the maxillary incisive canals were not significantly different with aging. A significant difference in sizes but not Hounsfield scale values was found between normal maxillary incisive canals and maxillary incisive canal cysts. Based on a cut-off of over 6 mm in the width of incisive canals, maxillary incisive canal cysts could not be appropriately diagnosed for subjects over 60 years of age. Over 60 years of age, maxillary incisive canal cysts could be appropriately diagnosed based on a cut-off of over 7.1 mm in width of incisive canals. When maxillary incisive canals of the hourglass types were seen on sagittal sections, significantly more patients had maxillary incisive canal cysts than other types.

**Conclusion:**

In coincidentally diagnosing asymptomatic incisive canal cysts on imaging, we should apply different cut-offs for the size of the maxillary incisive canal for patients over and under 60 years of age. Specifically, the cut-offs for the long axis of maxillary incisive canal cysts were 7.1 mm for patients over 60 years of age and 6.0 mm for those under 60 years of age. In addition, we should pay attention to wider canals with hourglass shapes as indicative of cystic change of maxillary incisive canals.

## Introduction

The maxillary incisive canals are the normal landmarks located in the midline of the anterior maxillary regions. The terminal branches of the descending palatine arteries and the nasopalatine nerves that go through them are distributed into the anterior regions, including the anterior teeth. The locations and configurations of the maxillary incisive canals are important in surgical procedures involving the anterior maxillary regions, such as dental implant treatment, extraction of a mesiodens, and cystectomy of radicular and nasopalatine cysts [1].

Maxillary incisive canal cysts are relatively common developmental cysts in the maxilla. Representative clinical findings of maxillary incisive canal cysts are palatal swelling, displacement of anterior teeth, sublabial swelling, and low-grade pain [2]. If these signs could be recognized, the patients could relatively easily and appropriately be diagnosed as having incisive canal cysts based on additional imaging characteristics. If not, for example, when we have coincidentally encountered larger maxillary incisive canals on imaging in patients with other lesions suspected in daily clinical practice, we have struggled to decide whether the maxillary incisive canals should be diagnosed as cysts. The normal diameter of incisive canals is generally considered to be under 6 mm; when it exceeds 6 mm, cystic change should be considered [3–5]. However, we have often seen larger maxillary incisive canals, such as over 6 mm, and, in our empirical experience, more often in elderly persons. Therefore, we hypothesized that the diameters of the maxillary incisive canals might change according to age. If so, the present criteria for maxillary incisive canal cysts based on a size of 6 mm would not necessarily be adequate to diagnose cystic changes. There have been few reports of differences of shapes, sizes, and Hounsfield scale values between normal and abnormal maxillary incisive canals [4, 5], because the spread of these computed tomography (CT) modalities has led to improved understanding of the complex anatomy of the maxilla and mandible in the last 10 years [6–9].

In the present study, diagnostic criteria for maxillary incisive canal cysts based on changes in normal maxillary incisive canals that occur with age in the Japanese population are proposed based on the imaging characteristics, such as the shapes, sizes, and Hounsfield scale values of normal maxillary incisive canals and of maxillary incisive canal cysts.

## Materials and methods

This study involved 220 normal subjects without maxillary incisive canal cysts (118 males, 102 females; age range 20–89 years; mean age 38.9 ± 28.6 years) and 40 patients (24 males, 16 females; age range 23–86 years; mean age 54.0 ± 25.9 years) with maxillary incisive canal cysts on multi-detector row (MD) CT seen in the Division of Oral and Maxillofacial Radiology in Kyushu Dental University Hospital between 2009 and 2017 who were retrospectively analyzed. The inclusion criteria of the present sample for normal subjects were the absence of any factor affecting the evaluation of the maxillary incisive canal on MDCT, and from among such subjects, those for the present study were selected at random. The representative diseases of the normal subjects were pericoronitis of a maxillary third molar, assessment for potential maxillary dentures, marginal and periapical periodontitis, and pulpitis. In addition, the whole maxillary incisive canals of normal subjects that could be appropriately and precisely visualized without metal artifacts on MDCT were used in the present study. However, subjects with suspected pathological lesions in the anterior maxillary regions including impacted teeth, previously reported history of craniofacial malformations or syndromes, and a previous history of trauma or surgery were excluded in the present study. All patients with maxillary incisive canal cysts on MDCT were included. However, patients with factors that could affect the evaluation of the maxillary incisive canal on MDCT were excluded. Approval of the present study was obtained from the institutional review board of Kyushu Dental University (No. 16–6).

The distributions of the normal subjects and patients in the present study are shown in Table 1. In particular, the shapes, sizes, anatomic variations, and Hounsfield scale values of maxillary incisive canals in the maxilla and the sizes and Hounsfield scale values of maxillary incisive canal cysts were analyzed. The Hounsfield scale is a quantitative scale for describing radiodensity, and the unit is the Hounsfield Unit (HU), which is also called the “CT number”.

**Table 1:** Distributions of subjects without maxillary incisive canal cysts and patients with maxillary incisive canal cysts by age and sex

Age group	Subjects		Patients	
	Male	Female	Male	Female
20s	32	33	6	2
30s	25	23	2	2
40s	16	11	1	1
50s	11	9	5	3
60s	11	9	6	2
70s	11	9	3	3
80s	12	8	1	3
Total	118	102	24	16

MDCT was performed with an Activion 16 (Toshiba Co. Ltd., Tokyo, Japan). MDCT images were taken with reconstruction images at 0.5 mm from the axial plane with 0.5 × 16-mm-thick contiguous sections at the level of the maxilla. Images were obtained with soft-tissue-target windows and bone-target windows by standard algorithms. For CT imaging, the following parameters for the CT scan were used: 120 kV, 200 mA (2D Auto mA), 1.0 s per tube rotation, slice thickness of 0.5 mm, field of view of 180 × 180 mm^2^, helical pitch of 11:1, 5.50 mm/rotation table speed, 70–180 mm coverage, and 10.0–20.0 s acquisition time. Prior to visualization of the sagittal views, CT images in the axial and coronal planes were also acquired as reference slices for each subject to be used as a locator for extent and angulation on sagittal views and for precise anatomical analysis. The midline palatal suture and maxillary incisive canal were identified on the axial plane. The anterior nasal spine and nasal septum were also identified on the coronal plane. The maxillary incisive maxillary canals on the appropriate sagittal view were visualized based on the midline palatal suture and the maxillary incisive canal on the axial view and the anterior nasal spine and the nasal septum on the coronal view.

The following were retrospectively examined on MDCT in subjects with normal maxillary incisive canals: 1) shapes; 2) directions; 3) courses; 4) anatomic variations; 5) sizes; and 6) Hounsfield scale values. At the same time, the following were retrospectively examined in patients with maxillary incisive canal cysts: 1) sizes; and 2) Hounsfield scale values.

**Fig. 1:**
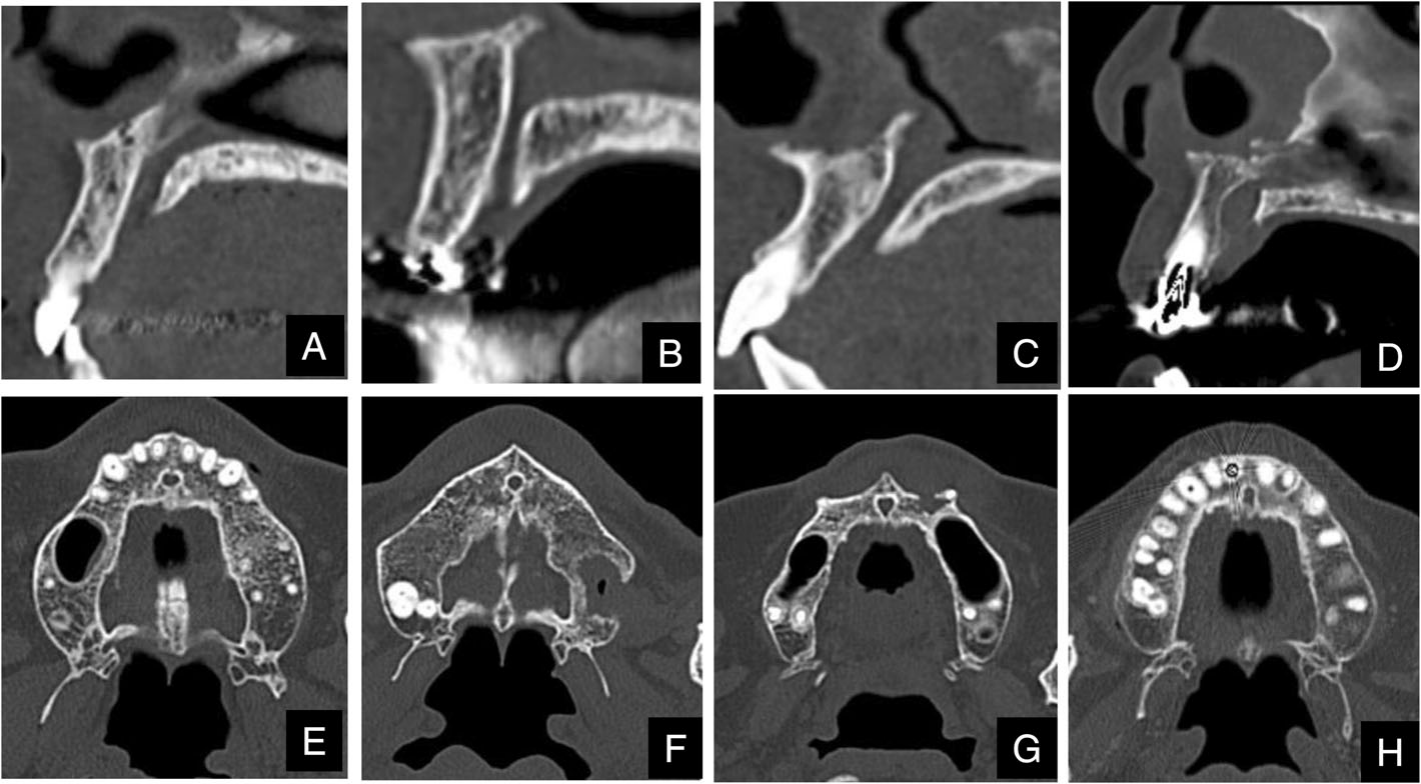
The shapes of normal maxillary incisive canals on CT divided into 4 categories. Based on CT sagittal sections of maxillary incisive canals, they are classified as cylindrical (**a**), funnel (**b**), spindle (**c**), or hourglass (**d**), and based on CT axial sections, they are round (**e**), oval (**f**), heart (**g**), or others (**h**)

**Fig. 2:**
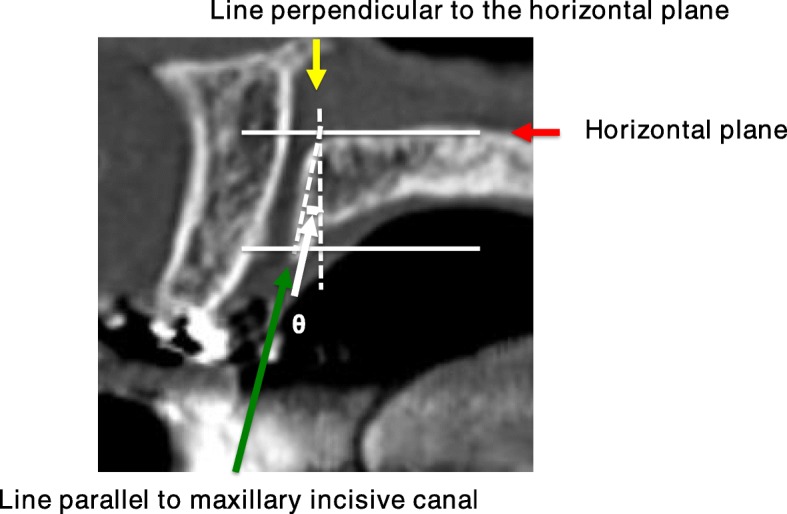
The directions and courses of the maxillary incisive canals on CT sagittal sections. The nasal floor is regarded as the “horizontal plane”. The three-dimensional reconstructions are re-oriented with reference to that plane. Based on the line perpendicular to the horizontal plane, the maxillary incisive canals whose course changes by > 10° from the vertical are considered “slanted“, and those whose course changes by < 10° from the vertical are considered “vertical”

**Fig. 3:**
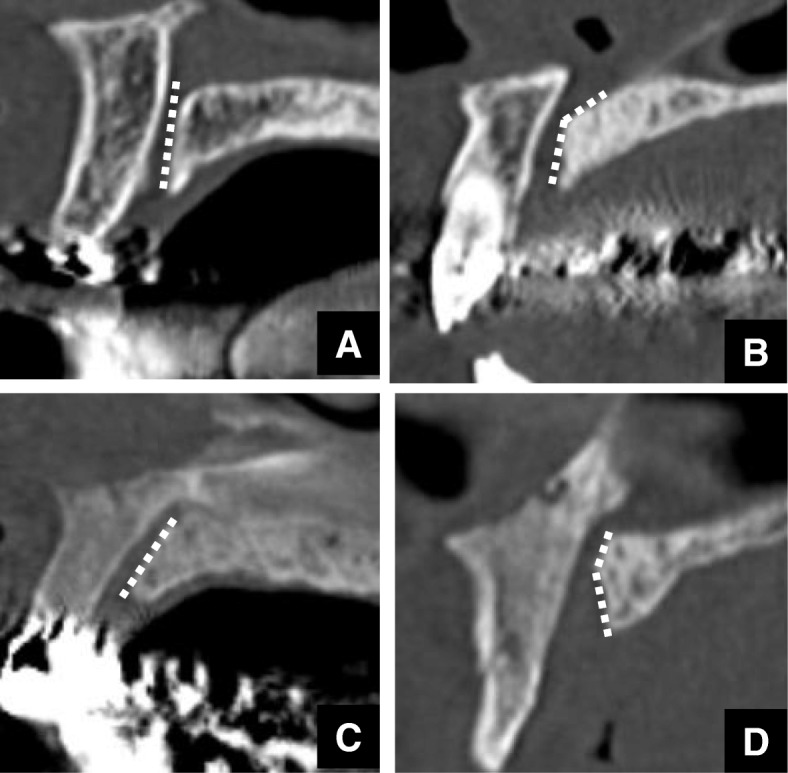
Whether the courses of the maxillary incisive canals are straight or curved is also noted based on the curvature of the palatal wall of the canals. Therefore, four types of incisive canals based on curvature are noted: vertical (**a**), vertical-curved (**b**), slanted (**c**), and slanted-curved (**d**). The slant angles of the maxillary incisive canals are the angles measured between the floors of the nasal fossa and the long axis of the maxillary incisive canals, which are considered to be the lines joining the midpoints of the antero-posterior diameters at the level of the hard palate

Shapes of normal maxillary incisive canals were divided into 4 categories according to Thakur et al. [5]. On sagittal sections of the maxillary incisive canals on CT, they were classified as cylindrical, funnel, spindle, or hourglass (Fig. 1a-d), and on axial sections, they were classified as round, oval, heart-based, or others except round, oval, or hourglass (Fig. 1e-h). In addition, the course, such as the angulation and curvature, of the maxillary incisive canals on sagittal views was investigated according to Thakur et al. (Figs. 2 and 3) [5]. The angulation in the course of the maxillary incisive canals is indicated in Fig. 2. The nasal floor was regarded as the “horizontal plane”. The three-dimensional reconstructions were re-oriented with reference to that plane. The line perpendicular was one line of the perpendicular plane to the horizontal plane, the line parallel was one line of the parallel plane to the maxillary incisive canal, and θ was the angle between the line perpendicular to the horizontal plane and the line parallel to the maxillary incisive canal. When the angle θ was changed by > 10° from the vertical, it was regarded as “slanted“, and those whose course changed by ≤10° from the vertical were regarded as “vertical” (Fig. 2). The curvatures in the course of the maxillary incisive canals are indicated in Fig. 3. Whether the curvatures of the maxillary incisive canals were straight or curved was also noted based on the curvatures of the palatal walls of the canals. Therefore, four types of maxillary incisive canals based on curvature were noted: vertical, vertical-curved, slanted, and slanted-curved. The slant angles of the maxillary incisive canals were the angles measured between the floors of the nasal fossa and the long axis of the maxillary incisive canals, which were considered to be the line joining the midpoints of the antero-posterior diameters at the level of the hard palate (Fig. 3).

**Fig. 4:**
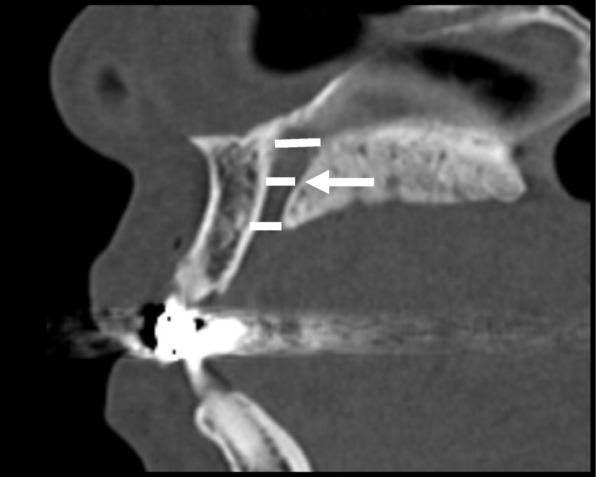
Based on CT sagittal sections of the incisive canals, the diameters of the nasal foramina are marked at the nasal entrances of the maxillary incisive canals, and the diameters of the incisive foramina are marked at the oral entrances of the canals. The canal diameters are measured at the midpoints between these two levels

Sizes of normal maxillary incisive canals were measured according to the methods of AI-Amery et al. [10]. In particular, they are illustrated on various points of measurement on a sagittal-cross section on CT (Fig. 4). Based on sagittal sections of maxillary incisive canals on CT, the diameters of the nasal foramina were measured at the nasal entrances of the maxillary incisive canals, and the diameters of the maxillary incisive foramina were measured at the oral entrances of the canals. The canal diameters were measured at the midpoints between these two levels.

**Fig. 5:**
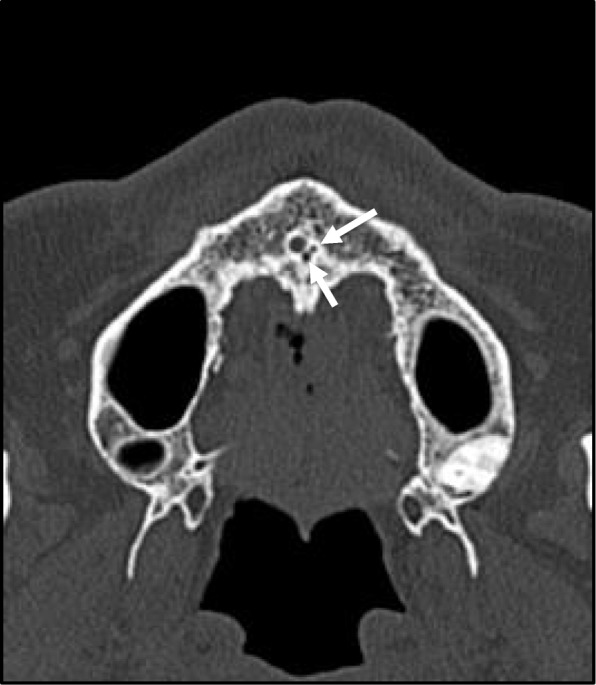
As anatomic variations, the number of openings at the nasal fossa of the maxillary incisive canals and the presence of accessory canals (arrow) in the maxilla are investigated

As anatomic variations, the numbers of openings at the nasal fossa in the maxillary incisive canals and the presence of accessory canals in the maxilla were investigated. With respect to the presence of accessory canals in the maxilla, the presence of two canals appearing bilaterally in the area of the lateral incisors and accessory canals in the region of the canine to the lateral incisor and extending to the alveolar crests was examined according to Eshak et al. (Fig. 5) [11].

**Fig. 6:**
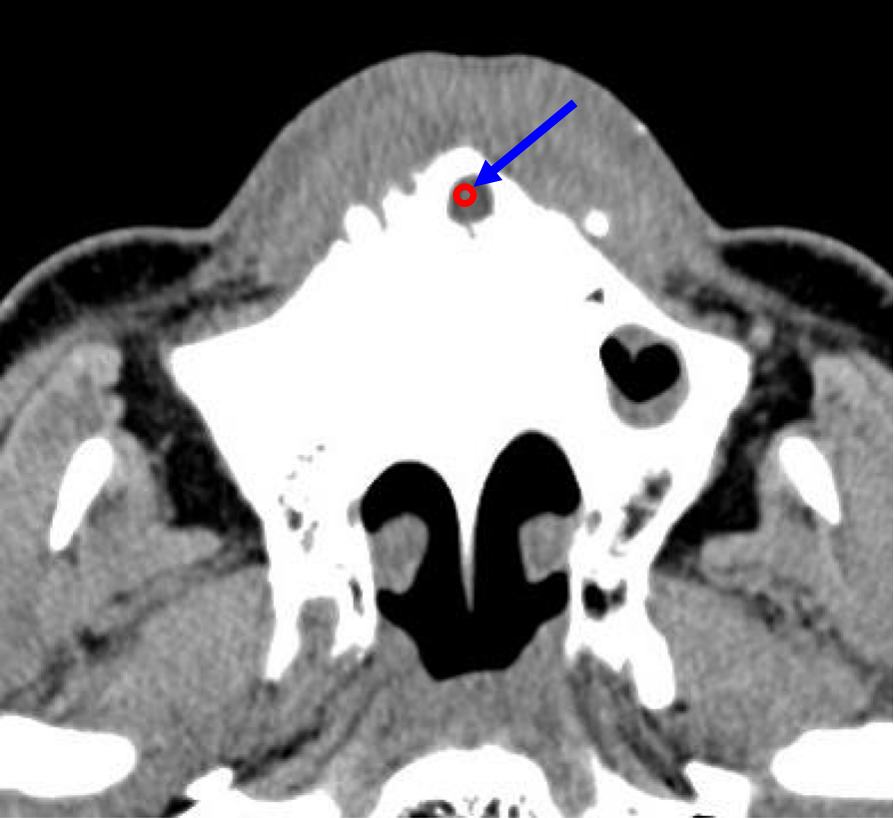
Hounsfield scale values within the incisive canal are measured in HU. ROIs are set and measured so that surrounding bones are not included

Hounsfield scale values within the incisive canal were also measured as Hounsfield Units (HU) (Fig. 6). The regions of interest (ROIs) were set and measured so that surrounding bones were not included.

The imaging examinations were independently evaluated by two radiologists (N.U., and T.T.) who assessed the various parameters mentioned above. Disagreements between examiners were discussed and resolved by consensus. Each observer performed two examinations with an interval of 1 week. When the evaluations were performed, the assessments from each observer were compared, and intra- and inter-observer agreements were calculated by the kappa test. The kappa analysis was performed before the disagreements among examiners were discussed and resolved. Intra-observer agreement for detection using the kappa values was 0.90. Inter-observer agreement for detection using the kappa values was 0.81.

All statistical analyses were performed using SPSS version 23 statistical software (SPSS, Chicago, IL, USA). Results were considered significant at *p* < 0.05. Categorical variables were compared by the chi-squared test. The significance of differences between continuous independent variables was evaluated with a nonparametric test (Mann-Whitney U test) if the data did not follow a normal distribution. Relationships between categorical variables were assessed using Pearson’s correlation coefficient. The specific cut-off values for diagnosing incisive canal cysts were estimated from receiver operating characteristic (ROC) curve analysis. Optimal cut-off values were chosen as the point on the ROC curve closest to the top left corner.

## Results

### Relationships of the distributions of shapes and angulation on CT in normal maxillary incisive canals with sex and age

The distributions of shapes in normal maxillary incisive canals on sagittal CT imaging are shown in Table 2, and those on axial CT imaging are shown in Table 3. No significant differences in the distributions of shapes in normal maxillary incisive canals on sagittal CT imaging were found between the sexes (Chi-squared test: *P* > 0.05) or among ages (Chi-squared test: *P* > 0.05). The results for axial sections were the same as those for sagittal sections for both sex and age (Chi-squared test: *P* > 0.05).

**Table 2:** Distribution of shapes on CT sagittal sections in normal and cystic maxillary incisive canals

Age group	Cylindrical			Funnel			Spindle			Hourglass		
	Male (Normal canals)	Female (Normal canals)	Patients (Cystic canals)	Male (Normal canals)	Female (Normal canals)	Patients (Cystic canals)	Male (Normal canals)	Female (Normal canals)	Patients (Cystic canals)	Male (Normal canals)	Female (Normal canals)	Patients (Cystic canals)
20s	12	13	1	13	13	1	4	3	1	3	4	5
30s	8	9	1	10	8	0	3	4	1	4	2	2
40s	5	4	1	4	2	0	4	2	0	3	3	1
50s	3	3	3	4	3	0	2	2	1	2	1	4
60s	3	3	2	2	3	0	1	1	1	5	2	5
70s	3	2	1	3	1	1	2	3	1	3	3	3
80s	4	3	1	4	3	0	2	0	0	2	2	3
Total	38	37	10	40	33	2	18	15	5	22	17	23

*CT* Computed tomography

**Table 3:** Distribution of shapes on CT axial sections in normal and cystic maxillary incisive canals

Age group	Oval			Round			Heart			Others		
	Male (Normal canals)	Female (Normal canals)	Patients (Cystic canals)	Male (Normal canals)	Female (Normal canals)	Patients (Cystic canals)	Male (Normal canals)	Female (Normal canals)	Patients (Cystic canals)	Male (Normal canals)	Female (Normal canals)	Patients (Cystic canals)
20s	13	12	2	11	13	3	6	5	2	2	3	1
30s	11	9	2	7	8	1	5	4	1	2	2	0
40s	3	3	2	6	3	0	4	3	0	3	2	0
50s	5	3	3	3	3	3	2	2	1	1	1	1
60s	2	3	5	3	3	2	3	3	0	3	0	1
70s	3	4	3	4	1	2	2	3	1	2	1	0
80s	5	3	1	3	3	2	2	0	1	2	2	0
Total	42	37	18	37	34	13	24	20	6	15	11	3

*CT* Computed tomography

The distributions of angulation in normal maxillary incisive canals of the subjects are shown in Table 4. No significant differences in the distributions of angulation in normal maxillary incisive canals were found between the sexes (Chi-squared test: *P* > 0.05) or among ages (Chi-squared test: *P* > 0.05).

**Table 4:** Distribution of directions on CT in the normal and cystic maxillary incisive canals

Age group	≤10°			> 10°		
	Male (Normal canals)	Female (Normal canals)	Patients (Cystic canals)	Male (Normal canals)	Female (Normal canals)	Patients (Cystic canals)
20s	10	11	2	22	22	6
30s	7	9	1	18	14	3
40s	5	3	1	11	8	1
50s	3	3	3	8	6	5
60s	4	3	4	7	6	4
70s	3	3	4	8	6	2
80s	4	4	2	8	4	2
Total	36	36	17	82	66	23

*CT* Computed tomography

The distributions of curvature in normal maxillary incisive canals are shown in Table 5. No significant differences in the distributions of the curvature of normal maxillary incisive canals were found between the sexes (Chi-squared test: *P* > 0.05: *P* > 0.05) or among ages (Chi-squared test: *P* > 0.05).

**Table 5:** Distribution of the courses on CT in the normal and cystic maxillary incisive canals

Age group	Vertical			Vertical-curved			Slanted			Slanted-curved		
	Male (Normal canals)	Female (Normal canals)	Patients (Cystic canals)	Male (Normal canals)	Female (Normal canals)	Patients (Cystic canals)	Male (Normal canals)	Female (Normal canals)	Patients (Cystic canals)	Male (Normal canals)	Female (Normal canals)	Patients (Cystic canals)
20s	15	12	4	11	12	2	4	5	1	2	4	1
30s	11	10	2	7	8	1	4	3	1	3	2	0
40s	5	4	2	6	4	0	3	1	0	2	2	0
50s	5	3	3	4	3	2	2	2	2	0	1	1
60s	4	3	5	5	3	2	1	2	0	1	1	1
70s	5	4	2	3	4	2	2	1	2	1	0	0
80s	5	3	2	4	3	1	2	0	1	1	2	0
Total	50	39	20	40	37	10	18	14	7	10	12	3

*CT*: Computed tomography

### Relationships of anatomic variations (number of openings of maxillary incisive canals at the nasal fossa) of the normal maxillary incisive canals with sex and age

The distributions of anatomic variations of normal maxillary incisive canals in the subjects were as follows. The number of openings of the normal maxillary incisive canals at the nasal fossa was 2.2 ± 0.5 (mean ± standard deviation (SD)) overall. Thirty-five of 220 (16%) subjects were found to have accessory canals. No significant differences in anatomic variations (number of openings of maxillary incisive canals at the nasal fossa) of normal maxillary incisive canals were found between the sexes (Mann-Whitney U test: *P* > 0.05) or among the ages (Pearson’s rank correlation coefficient: *r* = 0.02, *P* > 0.05).

### Relationships of sizes and Hounsfield scale values of the normal maxillary incisive canals with sex and age

The distributions of sizes of normal maxillary incisive canals in the subjects are shown in Table 6. The size of normal maxillary incisive canals was 4.8 ± 1.5 mm (mean ± SD) overall. The sizes of normal maxillary incisive canals were 5.3 ± 1.4 mm (mean ± SD) in males and 4.5 ± 1.6 mm (mean ± SD) in females; a significant difference was found between the sexes (Mann-Whitney U test: *P* < 0.05). The sizes of normal maxillary incisive canals were significantly larger with age (Pearson’s rank correlation coefficient: *r* = 0.55, *P* < 0.01).

**Table 6:** Distribution of sizes of normal maxillary incisive canals on CT

Age group	Male	Female
20s	4.3 ± 1.1	3.8 ± 1.4
30s	4.5 ± 1.2	4.2 ± 1.2
40s	4.9 ± 1.2	4.4 ± 0.9
50s	5.1 ± 1.2	4.7 ± 1.5
60s	5.6 ± 1.3	5.7 ± 1.5
70s	6.8 ± 2.0	6.2 ± 2.0
80s	6.6 ± 1.4	6.0 ± 2.3
Total	5.3 ± 1.4	4.5 ± 1.6

*CT* Computed tomography

The distributions of Hounsfield scale values in normal maxillary incisive canals in the subjects were as follows. The Hounsfield scale value in normal maxillary incisive canals was 16 ± 50 HU (mean ± SD) overall. The Hounsfield scale values within maxillary incisive canals were 18 ± 43 HU (mean ± SD) in males and 13 ± 58 HU (mean ± SD) in females. There were no significant differences in Hounsfield scale values in maxillary incisive canals between the sexes (Mann-Whitney U test: *P* > 0.05) or among the ages (Pearson’s rank correlation coefficient: *r* = 0.15, *P* > 0.05).

### Relationships of distributions of shapes and angulation on CT in incisive canal cysts with sex and age

No significant difference in the distributions of the shapes of maxillary incisive canals with cysts on sagittal CT imaging was found between the sexes (Chi-squared test: *P* > 0.05) and among the ages (Chi-squared test: *P* > 0.05). The results for axial sections were the same as those for sagittal sections for both sex and age (Chi-squared test: *P* > 0.05). A significant difference in the distributions of shapes was found between normal maxillary incisive canals and incisive canal cysts (Chi-squared test: *P* < 0.05). With maxillary incisive canals of hourglass types on sagittal sections, there were significantly more patients with maxillary incisive canal cysts than subjects with normal canals.

No significant difference in the distributions of angulation in the maxillary incisive canals of patients with cysts was found between the sexes (Chi-squared test: *P* > 0.05) or among the ages (Chi-squared test: *P* > 0.05). No significant difference in the distributions of angulation was found between normal maxillary incisive canals and incisive canal cysts (Chi-squared test: *P* > 0.05).

No significant difference in the distributions of the curvature of normal maxillary incisive canals of patients with cysts was found between the sexes (Chi-squared test: *P* > 0.05: *P* > 0.05) or among the ages (Chi-squared test: *P* > 0.05). No significant difference in the distributions of curvature was found between normal maxillary incisive canals and incisive canal cysts (Chi-squared test: *P* > 0.05).

### Relationships of sizes and Hounsfield scale values of incisive canal cysts with sex and age

The distributions of sizes of maxillary incisive canal cysts in the patients are shown in Table 7. The size of incisive canal cysts in patients was 9.3 ± 3.6 mm (mean ± SD). A significant difference in size was found between normal maxillary incisive canals and incisive canal cysts (Mann-Whitney U test: *P* < 0.05), but it was less between normal maxillary incisive canals of elderly subjects and cysts.

**Table 7:** Differences in the sizes on CT between normal maxillary incisive canals and incisive canal cysts

Age group	Subjects	Patients
20s	4.1 ± 1.2	8.8 ± 4.2
30s	4.3 ± 1.2	9.0 ± 1.8
40s	4.7 ± 1.2	9.3 ± 2.2
50s	4.9 ± 1.3	10.3 ± 3.3
60s	5.7 ± 1.4	9.1 ± 2.8
70s	6.5 ± 2.0	8.9 ± 4.0
80s	6.3 ± 1.9	8.8 ± 1.8
Total	4.8 ± 1.5	9.3 ± 3.6

*CT* Computed tomography

The Hounsfield scale values in incisive canal cysts in the patients are shown in Table 8. The Hounsfield scale value in incisive canal cysts was 29 ± 27 HU (mean ± SD). No significant difference in Hounsfield scale values was found between normal maxillary incisive canals and incisive canal cysts (Mann-Whitney U test: *P* > 0.05). The Hounsfield scale values of patients in their 30s and 40s were 63 ± 44 and 48 ± 47 HU (mean ± SD), respectively. The numbers were relatively higher than of other ages. However, no significant difference was found between Hounsfield scale values in patients in their 30s or 40s and in patients of other ages (Mann-Whitney U test: *P* > 0.05). At the same time, there was no significant difference in the Hounsfield scale values between patients and normal subjects in their 30s or 40s, respectively (Mann-Whitney U test: *P* > 0.05).

**Table 8:** Differences in the CT numbers between normal maxillary incisive canals and incisive canal cysts

Age group	Subjects	Patients
20s	12 ± 65	18 ± 25
30s	22 ± 53	63 ± 44
40s	18 ± 25	48 ± 47
50s	8 ± 45	19 ± 29
60s	15 ± 65	16 ± 10
70s	21 ± 38	15 ± 19
80s	10 ± 35	25 ± 22
Total	16 ± 50	24 ± 27

*CT* Computed tomography

### Relevance of age for diagnosing maxillary incisive canal cysts

**Fig. 7:**
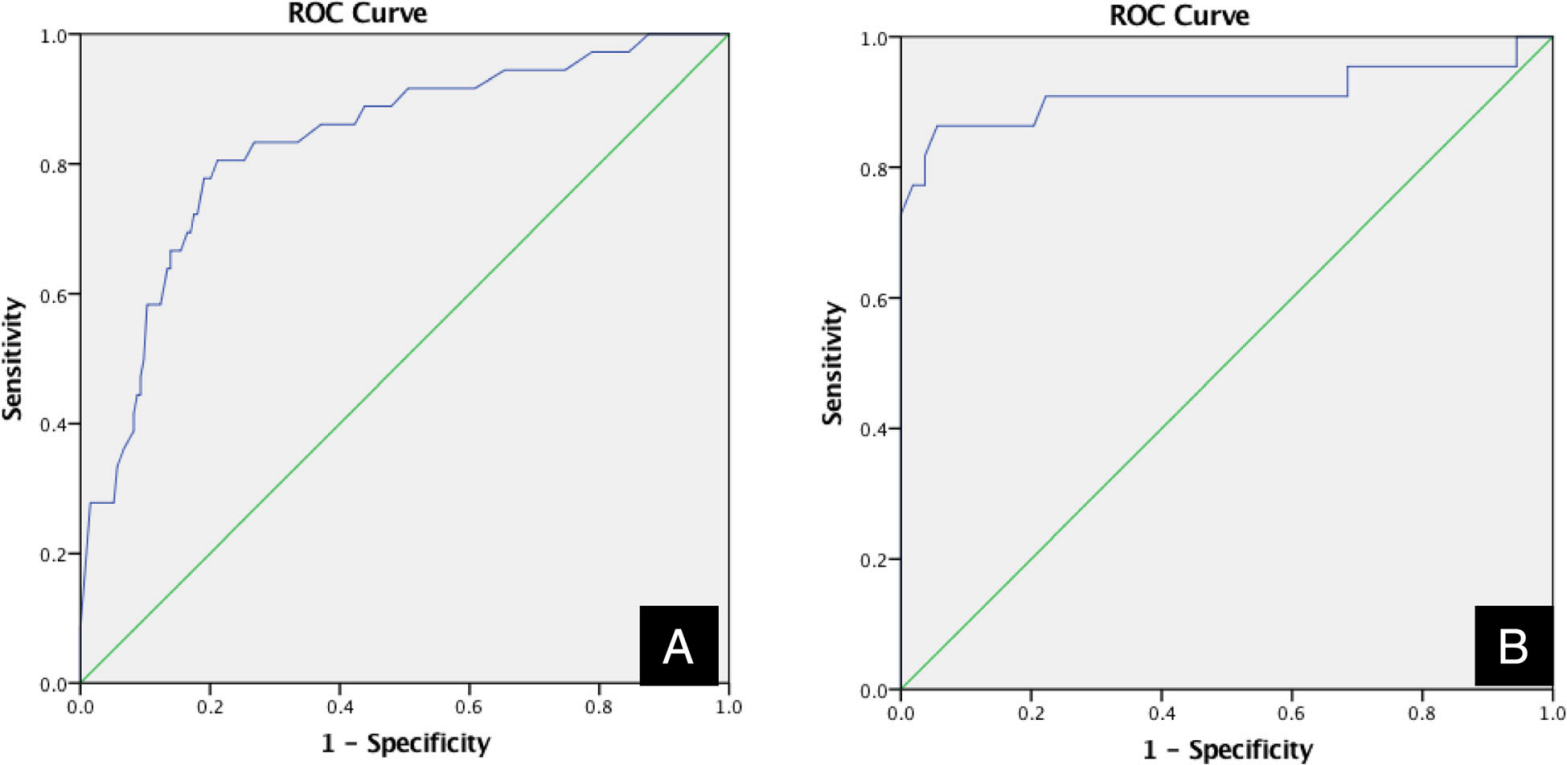
The cut-off values for diagnosing incisive canal cysts are estimated from ROC curves. **a**: cut-off point of age 60 years, sensitivity 66.7%, specificity 15.5%, area = 0.832, *P* < 0.001. **b**: cut-off point of size 7.1, sensitivity 90.1%, specificity 27.8%, area = 0.912, *P* < 0.001

The results from ROC analysis are shown in Fig. 7a. The ROC curve was used to determine the cut-off point for accurately diagnosing maxillary incisive canal cysts based on the axis of maxillary incisive canals. The ROC curve was created with parameters such as the age of subjects and the accuracy of maxillary incisive canal cysts. There was a statistically significant relationship for age: on the ROC curve for diagnosing incisive canal cysts, age was significant (0.832, *P* < 0.01,95% CI, 0.757–0.907), and the actual cut-off value of age for diagnosing incisive canal cysts was 60 years (0.667, 0.155).

### Proposed diagnostic criteria for maxillary incisive canal cysts for patients over or under 60 years of age

The cut-off point for diagnosis of maxillary incisive canal cysts should be examined in 60-year-old subjects from the ROC in Fig. 7b. The ROC curve was used to determine the cut-off point for accurate diagnosis of maxillary incisive canal cysts in the over 60-year-old subjects based on the axis of maxillary incisive canals. The ROC curve was created based on over 60-year-old subjects and the accuracy of maxillary incisive canal cysts. Next, the precision of using the long axis for appropriate diagnosis of maxillary incisive canal cysts in 60-year-old subjects based on the ROC curve was examined from the present data (0.912, *P* < 0.01,95% CI, 0.812–1.000). It was found that, if the long axis of the maxillary incisive canal was 7.1 mm for subjects over 60 years of age (0.901, 0.278), the diagnostic accuracy would be better than with other cut-offs (Fig. 7b). Thus, if the long axis of the maxillary incisive canal is over 7.1 mm in subjects over 60 years of age, it should be diagnosed as a maxillary incisive canal cyst, but if it is under, the diagnosis should be a normal maxillary incisive canal. If the new criteria were used based on the present data, the accuracy of differential diagnosis between normal and cystic changes might be about 90% in subjects over 60 years of age. On the other hand, the diagnostic criterion for the long axis of maxillary incisive canal cysts was 6.0 mm in subjects under 60 years of age (Fig. 7b). If the criterion were used based on the present data, the accuracy of differential diagnosis between normal and cystic changes might be about 90% in subjects under 60 years of age.

## Discussion

The most interesting result of the present study is that the sizes of normal maxillary incisive canals increased with age and that the common criterion for cystic changes (long axis exceeds 6 mm) of maxillary incisive canals would be relatively appropriate [3–5], except in the elderly population, but it is not necessarily valid and appropriate for the elderly population. New criteria for the exact differential diagnosis between normal maxillary incisive canals and cystic changes that take into account age are needed.

The other very interesting result is that the long axis for the appropriate diagnosis of maxillary incisive canal cysts in over 60-year-old subjects could be decided based on the present data. It was found that the cut-off value for the long axis of maxillary incisive canals or cysts of 60-year-old subjects was 7.1 mm. If the new criterion were to be used, based on the present data, the accuracy of differential diagnosis between normal and cystic changes might be about 90% because of the improvement of diagnostic accuracy of maxillary incisive canal cysts in over 60-year-old subjects. On the other hand, the cut-off for the long axis for distinguishing between maxillary incisive canal cysts and normal was 6.0 mm for under 60-year-old subjects [3–5]. Therefore, we suggest that the cut-off for the long axis of the maxillary incisive canal should be 7.1 for over 60-year-old subjects to distinguish between normal and cystic change in the maxillary incisive canal. The data using the two criteria mentioned above will now be gathered, and their validity needs to be evaluated.

The Hounsfield scale values and the sizes of maxillary incisive canals and cystic changes were also evaluated. Maxillary incisive canal cysts are indicated as a water-dense mass with a well-defined margin including the incisive canals [12, 13]. The normal maxillary incisive canals were also shown as water density in the present data. Certainly, no significant difference in Hounsfield scale values was found between the normal maxillary incisive canals and cysts. Therefore, the differential diagnosis between the normal maxillary incisive canals and cystic change canals could not be done by Hounsfield scale values based on the present data. The Hounsfield scale values of patients in their 30s and 40s were relatively higher than of those of other ages in the present data. However, there was no significant difference in Hounsfield scale values between patients and normal subjects in their 30s or 40s (Mann-Whitney U test: *P* > 0.05). The possible explanations were that the sample size of patients with maxillary incisive canal cysts was small, and the data could be affected by this limitation. If the Hounsfield scale values of maxillary incisive canals of patients in their 30s and/or 40s would be higher, cystic change could be inferred by doctors and dentists. Of course, as clinical findings, swelling and/or pain in the surrounding tissues could be detected in patients with maxillary incisive canal cysts. In addition, if cystic change with secondary infection in the maxillary incisive canals would occur, a well-circumscribed border with a thin rim of cortical bone and marginal sclerosis would disappear as a characteristic imaging finding [12, 13]. Thus, only the long axis as an imaging finding could be added as a new criterion for the differential diagnosis between normal and cystic change in the maxillary incisive canals.

Recently the locations, directions, shape in three dimensions, and so on of normal maxillary incisive canals have garnered much attention because the use of dental implant treatment has expanded widely in all of dentistry [14–16]. In particular, it was reported that the perforation of dental implants to maxillary incisive canals tended to occur in the elderly and edentulous patients because of the shorter distance from the maxillary incisive canal to the buccal cortical margin of alveolar bone occurring with maxillary frontal tooth loss [14]. Cone beam CT and MDCT are considered to be the most reliable and useful imaging modalities for examination of the maxilla and mandible, including the teeth, due to their ability to obtain high-quality images of bone tissue [6–9]. Together with the present results and reports by Jia et al., we speculated that one of the causes for perforation of dental implants to the maxillary incisive canals tending to occur in elderly and edentulous patients was related to the expansion of maxillary incisive canals, in addition to alveolar bone loss that accompanies tooth loss [15, 16]. Thus, dental implant treatments should be performed in the relatively early period after tooth extractions because of the two reasons mentioned above.

On the other hand, the distributions of locations, directions, and shapes in three dimensions of normal maxillary incisive canals were not found to change with age, and these data were similar to the previous studies [10]. Interesting information about the shapes of maxillary incisive canals related to cystic changes was discovered. Specifically, there were significantly more patients with maxillary incisive canal cysts than subjects with normal canals when maxillary incisive canals of hourglass types were seen on sagittal sections. The present result means that subjects with the maxillary incisive canals of hourglass types on sagittal sections might tend to occur the cystic change of maxillary incisive canal. Therefore, we should pay attention to wider canals with hourglass types of shapes to occur cystic change of maxillary incisive canals. However, no significant difference in distributions of angulations, or curvatures was found between normal maxillary incisive canals and incisive canal cysts. Therefore, the tendency to maxillary incisive canal cysts derived from normal canals could not be predicted based on morphologic analysis except shapes.

In the distribution of the number of openings in the maxillary incisive canals at the nasal fossa, two was most common, and subjects with 1, 2, 3, and 4 openings were found. However, there were no cases of 5 and 6 openings at the nasal fossa, unlike reports from India [5, 17–19]. The mean long axis in the present data was relatively short, also similar to the previous reports [4, 20]. The possible explanation is that the differences in the long axis and the number of openings between the present and previous data might reflect differences between races. The expansion of the maxillary incisive canals in elderly and edentulous patients was also noted in some reports [5, 15, 20]. In ethnic groups other than Japanese, expansion of maxillary incisive canals might commonly tend to occur after frontal tooth extractions. The distribution of shapes and directions of maxillary incisive canals did not change before and after frontal tooth extractions [10]. On examining the maxillary incisive canals using CT, accessory vessels were visualized in the maxilla around the incisive canals. Therefore, the presence of accessory vessels in the maxilla of Japanese persons was evaluated, and it was found that accessory canals in the maxilla were present in about 16%. In addition, the accessory canal presented as an accessory canal in the region of the canine to the lateral incisor extending to the alveolar crest of the maxilla, as in a previous report [11]. These reports mean that there are normal variations around the maxillary incisive canals, and that examination using CT is necessary to evaluate the maxilla in pre-treatment, including for dental implants.

The limitations of the present study include the uneven distribution of patients and normals. At the same time, one half of normal individuals were below age 40 years, whereas only 30% of the patients fell into this group. Moreover, the other limitation was that only one patient was present in some age groups. Another limitation is that the definition of subjects with normal maxillary incisive canals was based on the absence of clinical findings. If the sizes of the canals would be relatively larger, cystic change could not be ruled out. Thus, the results may not be accurate. Further cohort studies are needed to elucidate the precise meaning of the present results. Therefore, we are planning to start a longitudinal study of maxillary incisive canals. However, X-ray exposure is a very difficult ethical problem for such a study. Another limitation is that the sample size was not very large, and a further limitation of this study was that the patients were all Japanese. Therefore, the variables of age and sex by race could not be studied in this study sample.

## Conclusion

The purpose of the present study was to determine the imaging criteria for incisive canal cysts in maxilla. A total of 220 normal subjects and 40 patients with incisive canal cysts on MDCT were retrospectively analyzed, looking specifically at shapes, sizes, anatomic variations, Hounsfield scale values, and so on of incisive canals and incisive canal cysts. The results showed that the maxillary incisive canals were 4.8 ± 1.5 mm in size overall. The maxillary incisive canals were wider with age. However, there were no differences with age in shapes, Hounsfield scale values, and anatomic variations in the maxillary incisive canals. The sizes of maxillary incisive canal cysts were 9.3 ± 3.6 mm overall. A significant difference in sizes was found between normal maxillary incisive canals and cysts, but not between normal maxillary incisive canals of elderly subjects and cysts. Based on a cut-off width over 6 mm in incisive canals, incisive canal cysts were appropriately diagnosed, except in some elderly subjects. With maxillary incisive canals of hourglass types on sagittal sections, there were significantly more patients with maxillary incisive canal cysts than subjects with normal canals. No significant difference in Hounsfield scale values was found between normal maxillary incisive canals and cysts. In conclusion, in diagnosing incisive canal cysts, the sizes of the maxillary incisive canal used should take into account age: in patients over the age of 60 years, a long axis of 7.1 mm should be used as the cut-off for maxillary incisive canal cysts, while 6.0 mm should be the cut-off for patients under 60 years of age. In addition, we should pay attention to wider canals with hourglass types of shapes because they may indicate cystic change of maxillary incisive canals.

### Clinical significance

When diagnosing incisive canal cysts, we need to consider age and sex differences. In particular, in elderly (over 60 years of age) persons, it is necessary to use a larger size of maxillary incisive canal, beyond the conventional standard, as the criterion.

## Data Availability

For reasons of confidentiality, the data will only be shared in aggregate form as presented in the figures and tables.
